# Does the feedback of blood results in observational studies influence response and consent? A randomised study of the Understanding Society Innovation Panel

**DOI:** 10.1186/s12874-023-01948-y

**Published:** 2023-06-07

**Authors:** Michaela Benzeval, Alexandria Andrayas, Jan Mazza, Tarek Al Baghal, Jonathan Burton, Thomas F. Crossley, Meena Kumari

**Affiliations:** 1 Institute for Social and Economic Research, University of Essex, Colchester, UK; 2 School of Psychological Science, University of Bristol, Bristol, UK; 3 European University Institute, Fiesole, Italy

**Keywords:** Longitudinal study, Feedback, Response rates, Mode, Demography, Socioeconomic factors

## Abstract

**Background:**

While medical studies generally provide health feedback to participants, in observational studies this is not always the case due to logistical and financial difficulties, or concerns about changing observed behaviours. However, evidence suggests that lack of feedback may deter participants from providing biological samples. This paper investigates the effect of offering feedback of blood results on participation in biomeasure sample collection.

**Methods:**

Participants aged 16 and over from a longitudinal study – the Understanding Society Innovation Panel—were randomised to three arms – nurse interviewer, interviewer, web survey – and invited to participate in biomeasures data collection. Within each arm they were randomised to receive feedback of their blood results or not. For those interviewed by a nurse both venous and dried blood samples (DBS) were taken in the interview. For the other two arms, they were asked if they would be willing to take a sample, and if they agreed a DBS kit was left or sent to them so the participant could take their own sample and return it. Blood samples were analysed and, if in the feedback arms, participants were sent their total cholesterol and HbA1c results.

Response rates for feedback and non-feedback groups were compared: overall; in each arm of the study; by socio-demographic and health characteristics; and by previous study participation. Logistic regression models of providing a blood sample by feedback group and data collection approach controlling for confounders were calculated.

**Results:**

Overall 2162 (80.3% of individuals in responding households) took part in the survey; of those 1053 (48.7%) consented to provide a blood sample. Being offered feedback had little effect on overall participation but did increase consent to provide a blood sample (unadjusted OR 1.38; CI: 1.16–1.64). Controlling for participant characteristics, the effect of feedback was highest among web participants (1.55; 1.11–2.17), followed by interview participants (1.35; 0.99 –1.84) and then nurse interview participants (1.30; 0.89–1.92).

**Conclusions:**

Offering feedback of blood results increased willingness to give samples, especially for those taking part in a web survey.

## Background

It is good practice to feedback clinically relevant findings to study participants where the benefit outweighs any potential harm [1]. Moreover, it is increasingly recognised that even when results may not have clinical significance, participants may wish to know them to understand more about themselves [2]. In a number of empirical studies about why people take part in longitudinal health studies, among both existing participants and the general public, a key motivator is the provision of personalised health information [3–5]. When study participants themselves have been asked if they wish to receive feedback, the vast majority do. For example, in a qualitative follow up study of patients with diabetes who had received feedback as part of a trial, 99% said they wished to receive it [6]. In a general population cohort study, 95% of participants requested feedback when it was offered to them [7]. Equally, participants cite not receiving feedback as the reason they will not provide biological samples. In a previous wave of *Understanding Society*, which collected blood samples for storage and future measurement only, 12% of participants stated that their reason for not giving a blood sample was not receiving feedback, and the study had a lower rate of providing a blood sample than similar studies [8].

However, providing participants with their individual health results is challenging and costly to do well [1, 2]. There are sensitivities about feeding back clinically relevant data: participants can see it as a ‘free health check’ [7, 9], misunderstanding the nature of data being collected, which is generally analysed to research only standards and may not meet clinical standards [9]. If not understood by participants, this may lead to inappropriate decisions about their health in the future. Giving individual feedback, therefore, needs to be based on high quality and timely lab processes, should only be given if the medical benefit outweighs harm, should be done in ways that ensure participants do not over-interpret them, and ensures participants can seek appropriate advice if they are concerned [1, 2]. Ensuring such standards are achieved places considerable demands on the study team during data collection. In addition, for longitudinal observational studies, feeding back findings may change people’s behaviour [3, 4] altering the trajectories of measures over time [10].

What we do not know, however, is how feeding back clinically relevant findings may affect participation in longitudinal studies [11], although participants in one qualitative study said feedback would make them more likely to take part in future studies [6].

This paper investigates whether offering feedback from blood samples as part of an ongoing longitudinal study influenced participants’ willingness to give blood samples and to take part in the study overall, both at the wave the samples are taken and in the subsequent follow up wave. Secondly, it investigates whether feedback had a differential impact on consent to a blood sample in the different modes (web v interviewer v nurse interview) and based on the length of time a participant had been in the study. Finally, the paper explores whether different social and demographic groups respond differently to the offer of feedback.

## Methods and Data

The Understanding Society Innovation Panel (IP) was set up in 2008, as an annual household survey, designed for experimental and methodological research relevant to longitudinal studies [12]. The initial sample was a stratified clustered sample of households in Great Britain south of the Caledonian Canal designed to be representative of the general population. Participants aged 16 and over who have not died, moved out of Great Britain or withdrawn from the study are invited to take part annually. To maintain a reasonable sample size, refreshment samples, selected in a similar way, were added at waves 4, 7, 10 and 11 [12]. The 12^th^ wave (IP12), conducted in 2019, focused on the collection of biomeasures [13]. A full protocol is set out here [14], but in brief, households eligible for IP12 (*N* = 2,401) were randomly allocated to three equal sized arms (mode): nurse interview, interviewer and web survey. Within each arm, households were randomly allocated into two equal groups to receive feedback or not. All household members aged 16 years or older were eligible for interview, and initially sent an advance letter inviting them to take part in the mode to which their household was allocated. Information about feedback was included in the participant information sheet, sent in advance of the interviews and identical for each mode of invitation: ‘*During the interview we will ask you if you would like us to send you feedback on a couple of the lab results we will have obtained for you. If you would like feedback, we will provide this information once the labs have processed your sample’ *[15].

Informed consent was obtained at two stages of the data collection process. Participants were sent information leaflets about the study with the invitation letter and invited to complete online or in person. Consent was assumed if they complete the online survey or took part in the in-person interviews. For blood samples, written consent forms were provided and signed copies were returned with the blood samples.

After six weeks, those allocated to a web survey but who had not taken part were followed up by an interviewer. After 15 weeks those allocated to the nurse, and after 12.5 weeks those allocated to an interviewer, who had not taken part, were offered a web survey (some opted for this earlier) and were also issued to interviewers to offer a final telephone interview. The telephone protocol was based on the web survey. Fieldwork took place between 11^th^ July and 24^th^ November 2019. Most feedback letters were sent in December 2019. However, some batches were not due to be sent until 2020 and then the laboratories closed due to the pandemic. However, all feedback was sent by July 2020 before the start of the next wave. The subsequent wave of the IP (Wave 13) took place between 14th July and 11th November 2020; given the COVID-19 pandemic this wave was conducted by web or telephone only [12].

Within the IP12 interview, participants were invited to provide a range of biomeasures [14]. For this paper we focus on the request to provide a blood sample. Participants allocated to a nurse were asked to give both venous (VBS) and dried blood samples (DBS) during the interview. In both cases the nurse, trained in venepuncture, took the blood samples after seeking written consent from the participant and checking for contraindications. For the venous blood the nurse posted the sample to the lab, for the DBS, the DBS card was left with the participant to dry before they returned it. In the interviewer mode, the interviewer explained to participants about the DBS, and if they consented, left a kit for them to take their own sample and return it. In the web survey participants were asked if they would like to carry out a blood test themselves and if so, a DBS kit was sent to them. In all modes, for those participants living in households randomly allocated to the feedback group, once the bloods had been processed, they were sent their total cholesterol and HbAlc results, and were advised to consult their GP if their results were above recommended cut-offs. All participants received a £5 voucher for returning their samples.

Our first hypothesis was that those offered feedback would be more likely to take part in the study overall, both in the current and subsequent wave, and be more likely to consent to blood sample collection. The second hypothesis was that feedback would have a bigger impact on participation where the burden of collection for the participant was higher, and the encouragement/support lowest (i.e., the impact on web interviews would be greater than on interviewers’ ones, and impact on the latter would be greater than on nurse interview). This would obviously be truer when examining the mode the participants took part in than the mode to which they were randomly allocated. We hypothesized that the longer a participant had been in the study the less effect offering feedback would have on their participation, as they are already committed to the Study. We did not have specific hypotheses for how feedback would influence participation among different social groups.

We compared the response rate to the overall survey for those offered feedback or not in IP12 (the biomarker wave) and IP13 (the subsequent wave). We then investigated in more detail consent to give blood samples by feedback group: and by mode arm both issued (random mode allocation) and actual mode. The latter includes those participants who switched modes and is not therefore random, although as noted above most participants took part in the mode to which they were initially allocated. Actual mode may better reflect the burden and encouragement the participant may have received to take part in this aspect of the study.

We wished to assess whether participants were willing to give blood, rather than whether it was actually possible to provide a sample. Given the collection processes described above, the best way of capturing this was slightly different for each way of collecting the blood. For the VBS this was based on the consent question, whether or not a sample was actually collected by the nurse, but for the DBS samples this was based on whether the consent form and sample were returned (since these happened in tandem) to the Study team. For simplicity these are described as consenting to give a sample. In addition, in the descriptive tables we also report on whether participants requested a DBS kit (which might indicate willingness), but this is not a primary focus as often they did not then proceed to give blood.

Eight covariates were included in the models, all collected in the same data collection: sex (male v female); age (grouped as < 30; 30 to < 50; 50 to < 70; 70 +); education as measured by qualifications obtained (grouped as degree or equivalent; GCSE and A-levels or equivalent; no qualifications or ‘other’); self-assessed health (grouped excellent and good v fair and poor); use of NHS in last 12 months as a hospital or clinic out-patient (none v at least once); country of residence (England, Wales or Scotland); whether in paid employment (yes v no); and, length of time in study (original sample; joined between waves 4 and 10; joined at wave 11).

Descriptive analyses were carried out to explore basic differences in response rates by feedback group. Logistic regression models were calculated to examine the difference feedback made to providing blood samples. Four separate models were calculated – one for each mode and one for the combined sample. Covariates were added in two separate groups – participant characteristics and time in study – and then combined. In the overall model an interaction between mode and feedback was included. Complete case analysis was undertaken as item missingness was small (5%). Analysis was carried out in the statistical software R.

## Results

At IP12, 2401 households were eligible for inclusion, and at least one adult participated in 1408 (58.6%) households. Within participating households there were 2692 eligible adults and of these 2162 participants took part in a full interview (80.3%). The analytical sample is 2047 when cases with item missing data on the covariates are removed.

As shown in Table 1 most of the sample took part in their allocated mode. Only 32 participants initially assigned a nurse interview (4.5%) and 99 initially assigned an interviewer (15%) chose to take part in the web or telephone survey. While 209 participants initially allocated to the web-survey (26%) chose to be interviewed in person.

**Table 1:** Allocated and actual interview mode for the interviewed sample, IP12

	Allocated to Nurse	Allocated to interviewer	Allocated to Web	Total
Face-to-face interview by nurse	677	-	-	677
Face-to-face interview	-	562	209	771
Participated by web/telephone survey	32	99	583	714
Total	709	661	792	2162

At IP12 there was very little difference in the response rates overall between the group offered feedback in advance and those not offered feedback. Among eligible adults 80% (1117/1395) of those offered feedback took part, compared to 81% (1045/1297) in non-feedback households. A year later, in 2020, 2270 eligible IP12 participants were invited to take part in IP13. The IP13 response rate was marginally lower among those offered feedback in the previous wave (83.8%, *n* = 981/1171) than those not in the feedback group (84.5%, *n* = 929/1099).

Table 2 shows the sample characteristics of those who took part in IP12, comparing those allocated to the feedback and non-feedback groups. Overall, the characteristics of the feedback and non-feedback groups are very similar.

**Table 2:** Sample characteristics of IP12 participants in feedback and non-feedback arms

Characteristic	Non feedback	Feedback	Overall
Eligible households (N)	1204	1197	2401
Participating households (N, % of eligible)	691 (57%)	717 (60%)	1408 (59%)
Eligible individuals* (number within participating households) households)	1297	1395	2692
Adult full interviews* (N, percent eligible individuals)	1045 (81%)	1117 (80%)	2162 (80%)
**Characteristics of those interviewed (N, % total)**			
**Age group**			
< 30	151 (14%)	170 (15%)	321 (15%)
30–49	316 (30%)	290 (26%)	606 (28%)
50–69	410 (39%)	425 (38%)	835 (39%)
70 +	168 (16%)	232 (21%)	400 (19%)
**Sex**			
Male	493 (47%)	485 (43%)	978 (45%)
Female	552 (53%)	632 (57%)	1,184 (55%)
**Country**			
England	923 (88%)	990 (89%)	1,913 (88%)
Wales	55 (5.3%)	54 (4.8%)	109 (5.0%)
Scotland	65 (6.2%)	70 (6.3%)	135 (6.2%)
Missing	2 (0.2%)	3 (0.3%)	5 (0.2%)
**Highest qualification**			
Degree or higher	447 (43%)	460 (41%)	907 (42%)
A-level/GCSE	441 (42%)	465 (42%)	906 (42%)
Other/None	139 (13%)	165 (15%)	304 (14%)
Missing	18 (1.7%)	27 (2.4%)	45 (2.1%)
**In paid employment**			
Yes	574 (55%)	583 (52%)	1,157 (54%)
No	470 (45%)	530 (47%)	1,000 (46%)
Missing	1 (< 0.1%)	4 (0.4%)	5 (0.2%)
**Self-rated general health**			
Excellent/Good	750 (72%)	809 (72%)	1,559 (72%)
Fair/Poor	270 (26%)	273 (24%)	543 (25%)
Missing	25 (2.4%)	35 (3.1%)	60 (2.8%)
**Hospital or clinic outpatient last 12 months**			
No	564 (54%)	607 (54%)	1,171 (54%)
Yes	478 (46%)	502 (45%)	980 (45%)
Missing	3 (0.3%)	8 (0.7%)	11 (0.5%)
**Time in study**			
Original IP1 sample	381 (36%)	404 (36%)	785 (36%)
IP4/7/10 refreshment sample	461 (44%)	498 (45%)	959 (44%)
IP11 refreshment sample	203 (19%)	215 (19%)	418 (19%)

^*^excludes proxy interviews

Table 3 describes the blood consent rate by feedback group and the allocated and actual modes. Of those who participated in IP12 52% of those offered feedback consented to give a blood sample, while 45% of those who were not offered feedback consented to give a blood sample(p = 0.002). This varied by actual mode. Those interviewed by nurse were much more likely to consent to give blood (via venous or DBS) and offering feedback only made a modest 1% difference to their blood consent rates. For those interviewed by an interviewer, while a high percentage of the non -feedback group asked for a DBS kit to be left with them (80% non-feedback group; 76% feedback group), only 36% of the non-feedback group and 43% of the feedback group returned a blood sample. Finally, for those that took part through a web survey, more than double the number of participants requested DBS kits as actually gave samples, with kits being requested by 58% of the non-feedback group and 63% of the feedback group, and blood samples being returned by 27% of the non-feedback group and 35% of the feedback group. Comparing rates of consenting to give a blood sample by the initial randomly allocated mode, showed very similar patterns. Those allocated to a nurse were much more willing to give blood, with feedback having a very modest effect, unsurprising given few participants allocated to a nurse swapped mode. For those allocated to an interviewer, offering feedback increased the willingness to give a blood sample by 10 percentage points (48% v 38%), while on the web, 33% of the feedback group gave blood sample and only 27% of the non-feedback group.

**Table 3:** Blood consent rates by allocated and actual mode of interview and feedback group

**Characteristic:**		**Full Interview**	**Asked for DBS Kit**	**Consent to DBS**	**Consent to venous blood**	**Consent to either sample**
**Modes pooled**:						
	Overall	2162	1032	1036 (48%)		1052 (49%)
	Non-feedback	1045	499 (48%)	468 (45%)		473 (45%)
	Feedback	1117	533 (48%)	568 (51%)		579 (52%)
	t-test of feedback effect (p value)		0.99	0.005		0.002
**Allocated Mode:**						
i. Nurse	Non-feedback	334		243 (73%)	217 (65%)	248 (74%)
	Feedback	375		271 (72%)	256 (68%)	282 (75%)
ii. Interviewer	Non-feedback	309	238 (77%)	116 (38%)	-	
	Feedback	352	272 (77%)	170 (48%)	-	
iii. Web	Non-feedback	402	251 (62%)	109 (27%)	-	
	Feedback	390	256 (66%)	127 (33%)	-	
**Actual Mode:**						
i. Nurse	Non-feedback	319		240 (75%)	217 (68%)	245 (77%)
	Feedback	358		269 (75%)	256 (72%)	280 (78%)
ii. Interviewer	Non-feedback	361	289 (80%)	129 (36%)	-	
	Feedback	410	312 (76%)	178 (43%)	-	
iii. Web	Non-feedback	365	210 (58%)	99 (27%)	-	
	Feedback	349	221 (63%)	121 (35%)	-	

Table 4 shows the results of the logistic regression for consent to give a blood sample within each interview mode for both allocated and actual modes. With no covariates in the model, the feedback groups were more likely to give blood than the non-feedback groups. The top section of the table shows that this was highest in the group allocated to an interviewer (OR 1.57 (1.14 – 2.26)) and lowest among those allocated to a nurse (OR 1.26 (0.88 – 1.80)). Adjusting for participants characteristics or time in the Study made little difference to the impact of feedback on giving blood samples. The bottom section of Table 4 shows the association within the mode the participants actually took part. Again in all cases feedback groups had higher consent to give blood than those not offered feedback. The difference being highest for those who took part on the web. Adding covariates did not attenuate this. Comparing results between allocated and actual mode does reveal interesting comparisons. As might be expected, there was little difference in the effect of feedback between the allocated and actual mode for nurse interviews since 95% of those allocated to a nurse interview took part this way. However, there were differences for the other two modes. Feedback had less effect on those who actually took part with an interviewer than those allocated to one, and the opposite was true for the web; feedback had a bigger effect for those who took part by web than those allocated to it.

**Table 4:** Blood consent/provision rate by feedback group by allocated and actual mode, adjusting for IP12 characteristics

	**Nurse**			**Interviewer**			**Web**		
	**OR**	**95% CI**	**p**	**OR**	**95% CI**	**p**	**OR**	**95% CI**	**P**
**Allocated mode**									
**No controls**	1.26	0.88, 1.80	0.2	1.57	1.14, 2.16	0.005	1.38	1.01, 1.88	0.041
**Participant characteristics**	1.28	0.89, 1.84	0.2	1.53	1.10, 2.14	0.012	1.35	0.99, 1.86	0.062
**Study history**	1.25	0.87, 1.79	0.2	1.57	1.14, 2.16	0.006	1.38	1.01, 1.88	0.043
**N**	661			625			761		
**Actual mode**									
**No controls**	1.27	0.87, 1.86	0.2	1.40	1.04, 1.89	0.026	1.53	1.11, 2.12	0.010
**Participant characteristics**	1.30	0.89, 1.92	0.2	1.35	0.99, 1.84	0.055	1.55	1.11, 2.17	0.010
**Study history**	1.26	0.86, 1.85	0.2	1.40	1.04, 1.89	0.028	1.54	1.11, 2.13	0.009
**N**	633			718			696		
	**Nurse**			**Interviewer**			**Web**		

Table 5 shows the results of the logistic regression models for the full sample. With no covariates in the model those in the feedback group are 1.38 times more likely to give a blood sample, across the modes, than those who were not offered feedback. Actual mode, and switching mode were all significantly associated with giving blood. However, when the interactions between interview mode and feedback were included in the model the main effect for feedback became insignificant, reflecting the fact it only influenced participants willingness to give blood in the interviewer and web modes but not with a nurse. Adjusting for participant characteristics makes little differences to the odds ratio for being offered feedback. The only characteristic with a bigger impact on willingness to give blood was age, with those over 50 nearly twice as likely to give a blood sample as those under 30 years of age. Those with degrees or higher education (compared to those with less education) were also more likely to give blood. Time in the study had little impact on willingness to give a blood sample.

**Table 5:** Logistic regression models of consent to give blood, full sample, IP12

	**Mode of final interview**			**Mode and feedback interaction**			**Participant characteristics**			**Time in study**			**All covariates**		
**Characteristic**	**OR**	**95% CI**	**P**	**OR**	**95% CI**	**p**	**OR**	**95% CI**	**p**	**OR**	**95% CI**	**p**	**OR**	**95% CI**	**p**
**Feedback only, no covariates**	1.38	1.16, 1.64	< 0.001												
**Feedback**	1.42	1.17, 1.72	< 0.001	1.27	0.87, 1.86	0.2	1.38	1.15, 1.65	< 0.001	1.38	1.16, 1.64	< 0.001	1.27	0.86, 1.88	0.22
**Final mode of interview (Ref: Nurse)**															
Interviewer	0.21	0.16, 0.27	< 0.001	0.19	0.13, 0.26	< 0.001							0.19	0.13, 0.27	< 0.001
Web	0.13	0.10, 0.16	< 0.001	0.11	0.08, 0.16	< 0.001							0.11	0.08, 0.16	< 0.001
**Switcher dummy**	0.61	0.46,0.80	< 0.001				0.36	0.27, 0.46	< 0.001				0.62	0.46, 0.81	< 0.001
Feedback * Final mode of interview															
Feedback * Interviewer				1.10	0.68, 1.79	0.7							1.10	0.67, 1.80	0.71
Feedback * Web				1.20	0.73, 1.98	0.5							1.19	0.71, 1.98	0.51
**Age group (Ref: < 30)**															
30–50							1.32	0.99, 1.77	0.06				1.20	0.87, 1.65	0.27
50–70							1.99	1.50, 2.64	< 0.001				2.02	1.49, 2.75	< 0.001
70 +							2.03	1.42, 2.92	< 0.001				1.92	1.30, 2.84	0.001
**Sex (Ref: Male)**															
Female							1.11	0.92, 1.32	0.27				1.12	0.92, 1.36	0.25
**Country (Ref: England)**															
Wales							0.83	0.55, 1.26	0.39				0.98	0.63, 1.51	> 0.9
Scotland							1.13	0.79, 1.63	0.51				1.01	0.68, 1.50	> 0.9
**Highest qualification (Ref: Degree)**															
A-level/GCSE							0.83	0.68, 1.01	0.06				0.76	0.62, 0.94	0.01
Other/None							0.82	0.61, 1.10	0.19				0.81	0.59, 1.11	0.19
**In paid employment (Ref: Yes)**															
No							0.93	0.75, 1.16	0.51				0.90	0.71, 1.14	0.40
**Self-rated health (Ref: Excellent/Good)**															
Fair/Poor							0.99	0.80, 1.22	0.94				0.96	0.76, 1.21	0.72
**See NHS last 12 months (Ref: No)**															
Yes							1.09	0.90, 1.31	0.38				1.05	0.86, 1.28	0.65
**Time in study (Ref: Original IP1 sample)**															
IP4/7/10 refreshment										1.02	0.84, 1.24	0.9	1.04	0.84, 1.30	0.70
IP11 refreshment										1.04	0.81, 1.33	0.7	1.01	0.76, 1.33	> 0.9

In the nurse led interview, participants who refused were asked why they did not give blood. Across both the feedback and non-feedback groups the main reason for not consenting was dislike of giving blood (about half of respondents in both feedback groups cited this as their reason). The other main reason given was that they had recently had a blood test or health check.

## Discussion

In a longitudinal study, randomising offering feedback of blood samples to participants had a very small impact on their overall willingness to take part in the current or subsequent wave. However, it did have a significant impact on whether they gave a blood sample among the groups who took part via a web survey or with an interviewer, but not in those interviewed by a nurse. Older participants and those with more qualifications were also more likely to give blood than other groups.

We have found no other study that has empirically tested whether providing participants with feedback of their own health results influences their participation in the study. However, qualitative studies of participant’s views suggests that, while they would overwhelmingly like feedback, it does not affect their decision to take part in the study itself [6, 16]. Such studies have suggested that it may influence their willingness to take part in subsequent data collection [6], but we did not find this one year later. However, the uniqueness of 2020 because of the COVID-19 pandemic should be borne in mind.

With the increasing use of web surveys for data collection, evidence that feeding back results has the greatest impact on this group of participants is welcome. However, such forms of data collection place a greater burden on the study team to communicate effectively at all stages of the collection and feedback process as participants do not have contact with interviewers or nurses to answer queries and concerns. This study demonstrates that while interviewers did not collect samples themselves, they had higher rates of respondents requesting kits and later providing a blood sample, suggesting that the process of personal explanation and two-way communication may encourage participation. The highest rate of providing a blood sample and the least impact of offering feedback was among those interviewed by a nurse, which suggests nurse data collection may be sufficient without feedback. It may be that public respect and trust of nurses encourages all those willing and able to give blood to do so without any further incentive. There may be other ways of creating this trust without the use of nurse interviews, for example, having information in the web survey provided by medical practitioners or by having a nurse call participants to reassure them about the test and to encourage them to provide blood.

The differences in the impact of feedback between allocated and actual mode are interesting. Actual mode is a combination of those randomly allocated to the mode and those who choose to switch to it (web) or fail to take up the web survey and are followed up by an interviewer. This suggests that perhaps web participants are more personally motivated to engage with the survey, and feedback has the most impact on them. While adding those reluctant to take part by web to the interviewer group reduces the impact of feedback on providing a blood sample perhaps reflecting such participants are likely to be harder to engage more generally.

This study is the first to use a randomised design to investigate the effect of providing feedback on Study participation rates. It does, however, have some limitations. The sample size did not allow us to examine whether the effect of feedback varied for subgroups of the population beyond interview mode. As yet we do not know if the provision of feedback will change participants’ behaviours; qualitative research suggests it may [7], but the effect has not been quantified. However, we will be able to investigate this in future waves of the study.

## Conclusions

In a randomised study providing feedback of health-related findings did not impact on overall participation but did impact on the proportion providing blood samples, especially for those participating by web. Given that understanding population health, within its social context, has never been more important, identifying ways of doing this that ensures the highest participation rates, especially with remote forms of data collection, is vital. This study suggests ensuring participants are provided with health-related findings will have a positive impact on response.

## Data Availability

Data from the Understanding Society Innovation Panel used for this study are available for download from the UK Data Service SN: 6849, http://doi.org/10.5255/UKDA-SN-6849-14.

## Abbreviations

CI: Confidence Intervals
DBS: Dried Blood Spots
HbA1c: Glycosylated Haemoglobin
IP: Innovation Panel
MRC: Medical Research Council
OR: Odds Ratio

## References

[1] MRC and Wellcome Trust. Framework on the feedback of health-related findings in research, MRC March 2014 https://mrc.ukri.org/documents/pdf/mrc-wellcome-trust-framework-on-the-feedback-of-health-related-findings-in-researchpdf/ Accessed 4 Oct 2021.

[2] National Academies of Sciences, Engineering, and Medicine. Returning Individual Research Results to Participants: Guidance for a New Research Paradigm. 2018. Washington, DC: The National Academies Press. 10.17226/25094. Accessed from http://nap.edu/2509 10/4/2021. 30001048

[3] Hunter J, Corcoran K, Leeder S, Phelps K (2012). Appealing to altruism is not enough: motivators for participating in health services research. J Empir Res Hum Res Ethics.

[4] Dahlin-Ivanoff S, Sterner TR, Blennow K, Skoog I, Falk Erhag H (2019). Was it worth it? Older adults’ experiences of participating in a population-based cohort study – a focus group study. BMC Geriatr.

[5] Merz S, Jaehn P, Pischon T, Fischere B, Wirkner K, Rach S, Guenther K, Obi N, Holmberg C (2023). on behalf of the AdvanceGender Study Group Investigating people’s attitudes towards participating in longitudinal health research: an intersectionality-informed perspective. Int J Equity Health.

[6] McElfish PA, Purvis RS, Scott AJ, Haggard-Duff LK, Riklon S, Long CR. "The results are encouragements to make positive changes to be healthier:" qualitative evaluation of Marshallese participants' perceptions when receiving study results in a randomized control trial. Contemp Clin Trials Commun. 2020 Feb 19; 17:100543. 10.1016/j.conctc.2020.100543. 10.1016/j.conctc.2020.100543 PMC7044511 32140610

[7] Lorimer K, Gray CM, Hunt K, Wyke S, Anderson AS, Benzeval M (2011). Response to written feedback of clinical data within a longitudinal study: a qualitative study exploring the ethical implications. BMC Med Res Methodol.

[8] Kumari M, Benzeval M. Collecting biomarker data in longitudinal surveys. In: Lynn P, editor. Advances in Longitudinal Survey Methodology. Chichester: Wiley; 2021. p. 26–46.

[9] Mein G, Seale C, Rice H, Johal S, Ashcroft R, Ellison G, et al. Altruism and participation in longitudinal health research? Insights from the Whitehall II study. Soc Sci Med*. *2012;75(12):2345–52. 10.1016/j.socscimed.2012.09.006 23031604

[10] Wills AK, Lawlor DA, Matthews F, Aihie Sayer A, Bakra E, Ben Shlomo Y, Benzeval M, Brunner E, Cooper R, Kivimaki M, Kuh D, Muniz-Terrera G, Hardy R (2011). Lifecourse trajectories of systolic blood pressure using longitudinal data from eight UK cohorts Plos Medicine.

[11] Booker CL, Harding S, Benzeval M (2011). A systematic review of the effect of retention methods in population-based cohort studies. BMC Public Health.

[12] Institute for Social and Economic Research. Understanding society – the UK household longitudinal study, innovation panel, waves 1–13, user manual. Colchester: University of Essex; 2021. http://doc.ukdataservice.ac.uk/doc/6849/mrdoc/pdf/6849_ip_waves_1-13_user_manual_oct_2021.pdf.

[13] University of Essex, Institute for Social and Economic Research. Understanding Society: Innovation Panel, Waves 1-13, 2008-2020. [data collection]. 11th Edition. 2021. UK Data Service. SN: 6849. 10.5255/UKDA-SN-6849-14.

[14] Al Baghal T, Benzeval M, Burton J, Crossley TF, Kumari M, Rajatileka S. Collection of biomarkers using nurses, interviewers, and participants: the design of IP12, understanding society working paper 2021–06. Colchester: University of Essex; 2021. https://www.understandingsociety.ac.uk/research/publications/546970.

[15] University of Essex, Institute for Social and Economic Research (2019) ‘ 57a Understanding Society Health IP_Health Measures_PIS_Nurse_F_v1 3 June 2019, 57d.Understanding Society Health IP_Health Measures_PIS_Web_N_v1, 57e.Understanding Society Health IP Health Measures_PIS_Interview_F_v1 3 June 2019’ https://www.understandingsociety.ac.uk/sites/default/files/downloads/documentation/innovation-panel/fieldwork-documents/wave-13/IP13-advance-mailing.pdf, downloaded 05/02/2023.

[16] Dixon-Woods M, Jackson C, Windridge KC, Kenyon S (2006). Receiving a summary of the results of a trial: qualitative study of participants’ views. BMJ.

